# High-density microdroplet cultivation reveals the essential role of microbial interactions in the growth of environmental microbes

**DOI:** 10.1128/mbio.03953-25

**Published:** 2026-04-10

**Authors:** Eun-Young Seo, Rikuta Suzuki, Yuki Takagi, Tomonori Kindaichi, Akiyoshi Ohashi, Setsu Kato, Yutaka Nakashimada, Yoshiteru Aoi

**Affiliations:** 1 Department of Molecular Biotechnology, Graduate School of Advanced Sciences of Matter, Hiroshima University, Hiroshima, Japan; 2 Ningbo Institute of Marine Medicine, Peking University, Ningbo, China; 3 Graduate School of Integrated Sciences for Life, Hiroshima University, Hiroshima, Japan; 4 Graduate School of Advanced Science and Engineering, Hiroshima University, Hiroshima, Japan; 5 Seto Inland Sea Carbon-neutral Research Center, Hiroshima, Japan; The Forsyth Institute, Somerville, Massachusetts, USA

**Keywords:** cultivation method, gel microdroplet, colony formation, microcolonies, high cell density, uncultures

## Abstract

The significance of microbial interactions in nature is not well understood because of technical limitations in observing how individual microbial cells begin to grow within complex microbial community. To address this limitation for further solution of microbial uncultivability, we developed a gel microdroplet aggregate in oil (GMD-agg) cultivation method. This method provides a high cell density (>10^8^ cells/mL) but maintains pure cultures of each growth unit. Millions of hydrogel particles (10–30 µm in diameter) entrapping single cells with medium softly aggregated in oil is the key feature of this method. In this study, to assess the effect of microbial interactions on the cultivation of environmental microorganisms, soil and activated sludge samples were cultured, and colony formation and diversity during cultivation in GMDs were investigated. The results showed that the cultivation efficiency of GMD-agg was approximately 10 times higher than that of the method in which microbial interactions were canceled. In addition, the growth of taxonomies containing many uncultured microorganisms, such as *Verrucomicrobia*, *Planctomycetia*, *Acidobacteria*, and *Vicinimibacteria*, was observed. Furthermore, when isolated strains were co-cultured at high cell densities with either inter- or intra-species, we observed a higher cultivation efficiency than when microbial interactions were canceled. Rebuilding growth-promoting microbial interactions using isolated strains demonstrated that microbial interactions positively influence microbial growth. These findings indicate the effectiveness of this cultivation method, revealing the crucial role of microbial interactions in the growth of environmental microorganisms and contributing to solving the issue of microbial uncultivability.

**IMPORTANCE:**

The overwhelming majority of environmental microbes remain uncultured, limiting our understanding of their physiology and ecological roles. Although microbial interactions have been predicted as one of the key factors for the growth of uncultivable microbial types, the effect of these interactions on cultivability remains poorly understood. In this study, we developed a new droplet-based co-cultivation approach that promotes microbial interactions while maintaining pure cultures and enables growth tracking at the single-cell level. This method significantly improved cultivability (approximately 10 times), including the growth of taxa that are difficult to cultivate. Direct observation of microbial growth in the community using this method clearly demonstrated that microbial interactions are essential for the growth of diverse microbial types. These findings underscore the importance of microbial interactions in cultivation and offer a basis for radically expanding microbial bioresources, manipulating microbial communities, and exploring previously unrecognized microbial interactions.

## INTRODUCTION

The majority of microorganisms are known to be difficult to cultivate. Colony formation on solid media, such as agar plates, which are widely used as the primary cultivation technique, remains a foundational method for isolating environmental microbes. However, a substantial discrepancy often exists between the number of microbial cells inoculated from environmental samples and the number of colonies that subsequently form on artificial media (1). This long-standing issue, known as “the great plate count anomaly” (2, 3), was recognized over a century ago (4) and remains unresolved. A similar discrepancy is observed between the vast diversity of uncultivated microbial species revealed by cultivation-independent methods, such as 16S rRNA gene analysis and single-cell or meta-genomics, and the comparatively limited number of cultivated representatives (5–10). This limitation poses a major bottleneck in advancing our understanding of microbial physiology, ecology, and their potential applications. Addressing this issue requires the development of novel cultivation strategies and a deeper understanding of why many microorganisms resist cultivation under laboratory conditions.

In environments with high cell densities, such as soil, interactions among microbes are thought to regulate both growth and community structure (11–15). Indeed, some microorganisms depend on microbial interactions for growth, relying on compounds supplied by “helper” organisms (16–20). However, standard isolation procedures are designed to obtain pure cultures and typically prevent these interactions. For instance, only a limited number of cells (up to 10⁴ cells per plate) can be inoculated on agar plates to maintain sufficient spacing, thereby preventing colony overlap and cross-contamination. This separation disrupts potential interactions, especially those mediated by diffusible metabolites, eliminating many growth-supporting relationships (21). Consequently, microorganisms that require such interactions have largely been overlooked by conventional cultivation methods, except in rare or accidental cases. Consequently, the true extent to which microbial growth depends on these interactions remains largely unclear.

In contrast, *in situ* cultivation techniques, where microorganisms are grown within their natural environment using membrane-bound devices, have enabled the recovery of previously uncultivated taxa (22–27). These methods aim to incorporate environmental growth factors that are absent in standard artificial media. While some of these factors likely originate from microbial neighbors, they also include other abiotic components. The specific contribution of microbial interactions, particularly via exchanged chemical signals or metabolites, to cultivability remains poorly understood, as isolating these variables experimentally is technically challenging. Traditional cultivation methods are not suited to maintaining the close spatial proximity needed for such interactions to occur via diffusion (28–30). However, increasing both inoculum size and spatial proximity conflicts with obtaining pure cultures and distinguishable colonies creates a fundamental limitation in conventional methods. Here, “inoculum size” refers to the quantity of microbial cells introduced initially into the culture, such as the number of cells present on an agar plate.

In this study, we developed a new microbial cultivation platform using microdroplet technology. The key structure of this approach is hydrogel particles, gel microdroplets (GMDs) measuring 10–30 µm in diameter, which encapsulate single cells with a medium softly aggregated within oil. This system supports extremely high cell densities (>10⁷–10⁸ cells/mL), comparable to those in soil (31), while still preserving isolation of individual cells or microcolonies. This enables microbial interactions to occur at spatial scales similar to those in natural environments, particularly in soils and biofilms. Crucially, the droplet-based confinement allows for the separate cultivation and tracking of individual colonies without risk of cross-contamination. We applied this method to environmental samples from soil and activated sludge to assess the influence of microbial interactions on cultivation efficiency. Furthermore, we analyzed the isolates recovered using this platform and demonstrated that both intra- and inter-species interactions significantly contributed to their growth.

## RESULTS

### Rationale for the new cultivation method to facilitate microbial interactions

**Fig 1:**
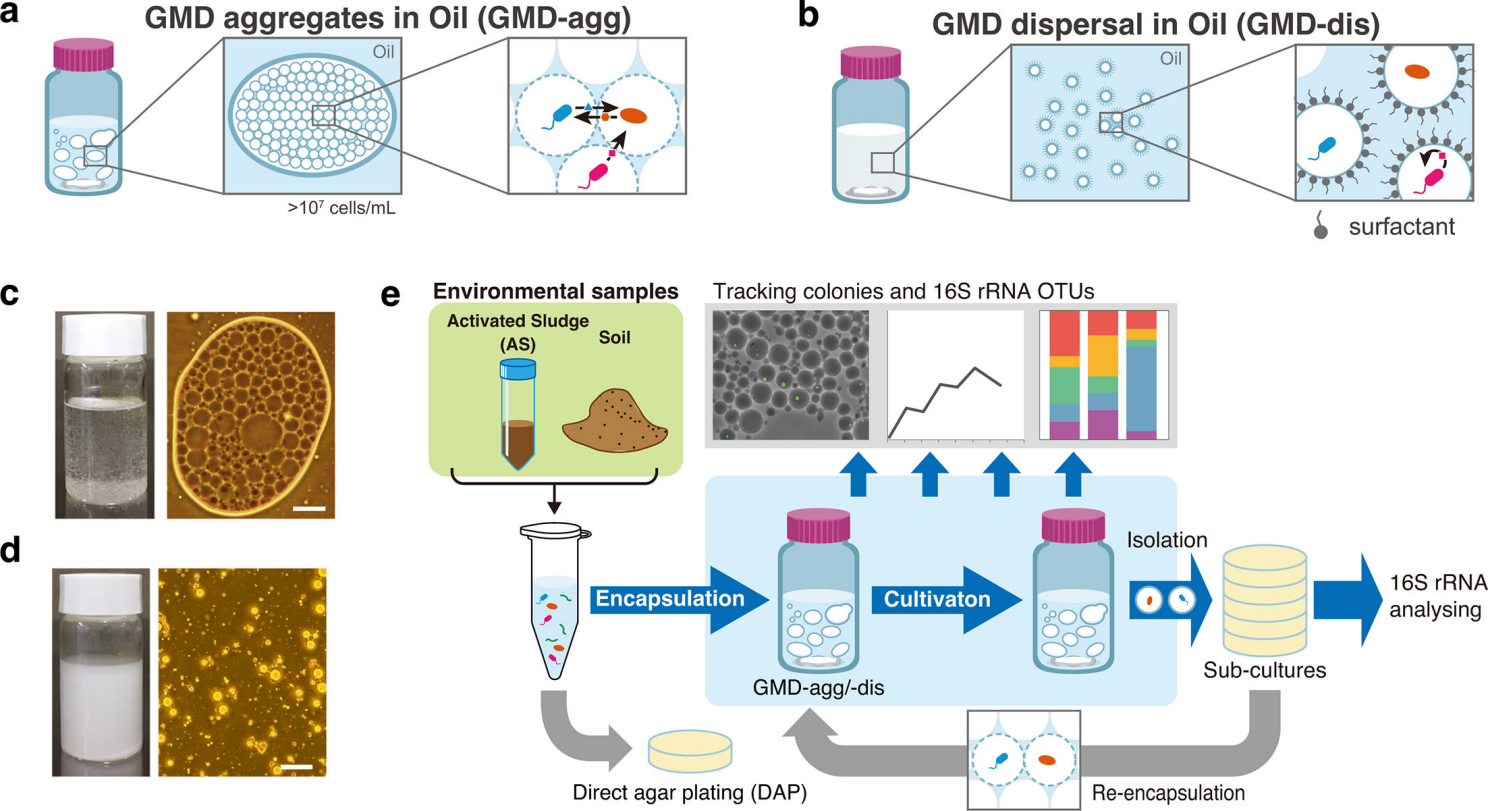
Conceptual overview of the GMD-agg cultivation method and experimental design. (**a**) Schematic representation of the gel microdroplet aggregation (GMD-agg) cultivation method. In this system, a large number (>10⁸) of gel microdroplets (10–30 μm in diameter), each containing a single microbial cell, are softly aggregated in oil. This structure enables the parallel cultivation of spatially separated microcultures, allowing water-soluble compounds to diffuse between droplets and facilitate microbial interactions while maintaining physical isolation between cultures. (**b**) Schematic representation of the gel microdroplet dispersion (GMD-dis) cultivation method. In contrast to GMD-agg, a stable emulsion is maintained, and droplets remain uniformly dispersed. Each droplet functions as a closed and independent culture unit. (**c**) Left: macroscopic view of the GMD-agg cultivation. Right: phase-contrast microscopic image of GMD-agg showing aggregated droplets in mineral oil. Scale bar, 50 μm. (**d**) Left: macroscopic view of the GMD-dis cultivation. Right: phase-contrast microscopic image of GMD-dis showing dispersed droplets in mineral oil containing 1% non-ionic surfactant Span 80. Scale bar, 50 μm. (**e**) Overview of the experimental demonstration of the new cultivation method (GMD-agg). Details are provided in Materials and Methods.

We developed a new cultivation method, termed “gel microdroplet aggregate in oil” (GMD-agg), which enables exceptionally high cell densities (>10⁷–10⁸ cells/mL) at the start of cultivation (Fig. 1a).

GMDs were prepared by emulsifying a hydrogel (agarose) containing both a medium and suspended microbial cells in oil with a surfactant. The resulting GMDs were then transferred to mineral oil without surfactants. In the absence of surfactants, hydrophilic GMDs aggregate within the oil phase. To maintain uniform oxygen availability (aerobic conditions) and promote consistent interactions, the GMD-oil mixture was gently stirred throughout incubation. This produced some small GMD-aggregates approximately 1–10 mm in diameter (Fig. 1c), which repeatedly came into contact with and separated from each other during cultivation. Therefore, through aggregation-to-aggregation connection events, any aggregate can obtain soluble products from any other GMD aggregate. In contrast, conventional droplet-based cultivation methods maintain each droplet in a fully emulsified and dispersed state (32–34), thereby limiting chemical diffusion between droplets (Fig. 1b and d).

In this study, we first confirmed the core concept of the GMD-agg method using a model microorganism. We then compared the cultivation efficiency of GMD-agg with that of two other methods: GMD cultivation without aggregation (GMD-dis) and conventional direct agar plating (DAP). Specifically, we focused on (i) the colony formation efficiency, (ii) microbial diversity and its changes during incubation, and (iii) the isolation of previously uncultivated microorganisms through subculturing from GMDs to agar plates (Fig. 1e).

### Demonstration of spatial separation and compound diffusion

A key feature of the GMD-agg method is that cells can exchange water-soluble compounds through diffusion without cross-contamination while maintaining a high cell density. We demonstrated this concept using *Escherichia coli* as a model organism. First, we confirmed that no cross-contamination between colonies was observed when cells were grown; in other words, cells do not move between GMDs. GMDs containing *E. coli* were mixed with empty GMDs and cultured under GMD-agg conditions. After 375 h of incubation, 1,000 of the initially cell-free GMDs were observed, and none showed growth (Fig. S1), confirming that colony separation was maintained and that there was no cross-contamination. We also tested the diffusion of water-soluble compounds between GMDs. GMDs containing *E. coli* were mixed with GMDs containing sodium azide (NaN_3_), a known growth inhibitor of *E. coli* (35), and were cultivated under GMD-agg conditions. After 20 h, growth inhibition was observed in *E. coli* containing GMDs, indicating that sodium azide had diffused between droplets (Fig. S2). These findings confirm that the GMD-agg method allows both spatial separation of growing colonies and the diffusion of soluble compounds across droplets.

### GMD-agg improves the cultivation efficiency for environmental samples

#### Improving the colony formation efficiency

To investigate the impact of the GMD-agg method on cultivation efficiency when using soil and activated sludge samples, we quantified the number of GMDs in which a microcolony formed during incubation. For comparison, the same samples were cultured using the GMD-dis and DAP methods. The number of GMDs containing microcolonies was counted by fluorescence microscopy, and the colony formation efficiency was calculated as the proportion of microcolonies relative to the number of inoculated cells on the first day of cultivation (see Materials and Methods for details). For the DAP method, plating efficiency was calculated as the proportion of visible colonies observed at the time of observation relative to the number of inoculated cells (visible colonies/inoculated cells).

**Fig 2:**
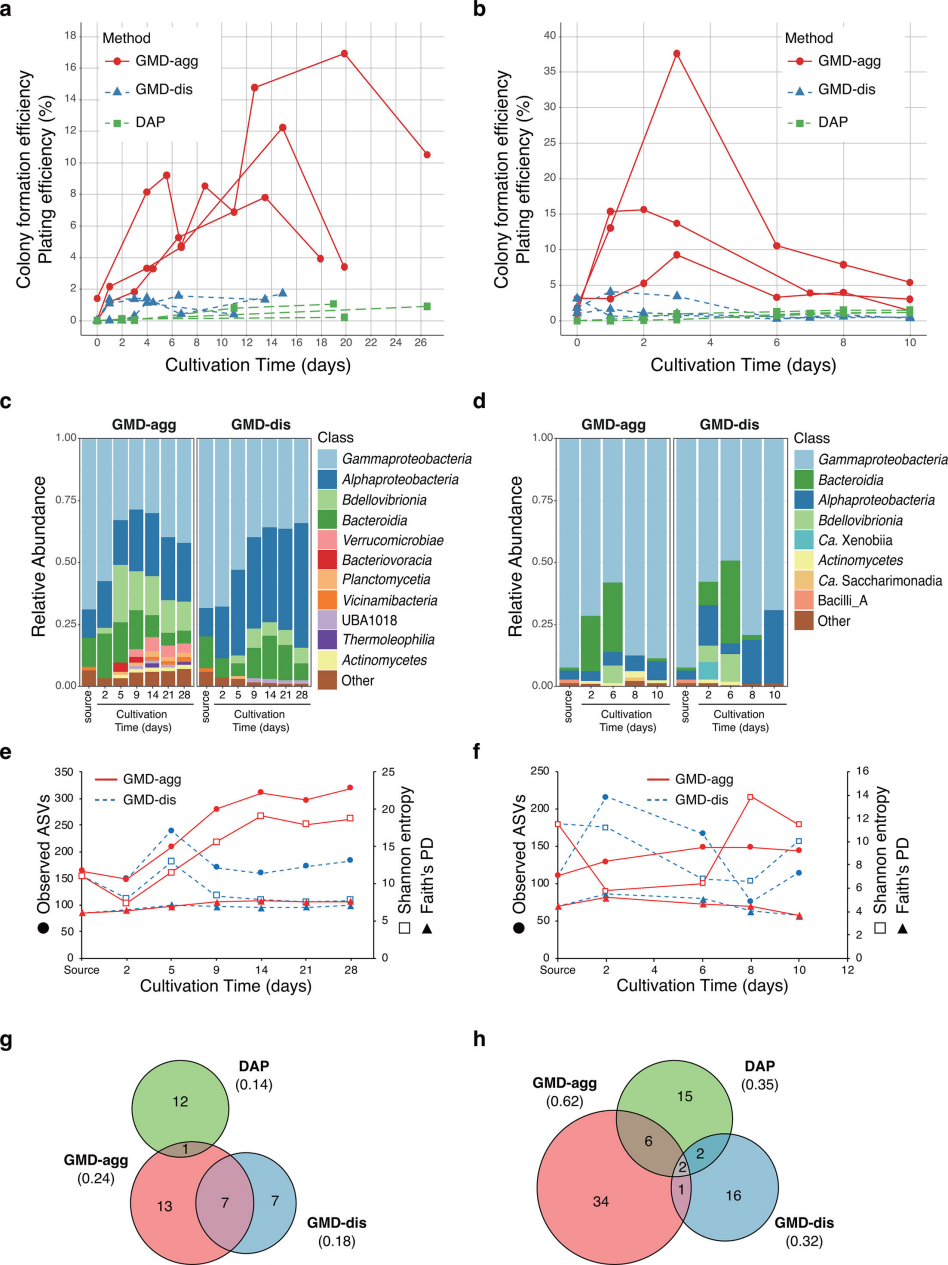
Comparison of cultivability and taxonomic composition under GMD-agg and GMD-dis cultivation conditions. (**a, b**) Tracking of colony formation over time in soil (**a**) and activated sludge (**b**) samples. Red circles and lines represent GMD aggregation in oil (GMD-agg), blue triangles and lines represent GMD dispersion in oil (GMD-dis), and green squares represent standard agar plate cultivation (DAP). (**c, d**) Microbial community dynamics during the cultivation of soil (**c**) and activated sludge (**d**) samples. The results of GMD-agg are shown on the left, and those of GMD-dis are shown on the right. ASVs with a relative abundance below 1% were grouped as “other.” “Source” indicates the inoculum source. (**e and f**) Alpha-diversity of GMD samples and inoculated sources from soil (**e**) and activated sludge (**f**). Red lines indicate GMD-agg, and blue dashed lines indicate GMD-dis. Dots represent observed ASVs, open squares denote Shannon entropy, and triangles represent Faith’s PD. (**g and h**) Venn diagrams showing the number of species uniquely or commonly isolated by each method (GMD-agg, GMD-dis, and DAP) from soil (**g**) and activated sludge (**h**) samples. The numbers in parentheses indicate the proportion of species relative to the total number of isolates for each method (see Tables S1 and S2).

Cells from the environmental sample were encapsulated into GMDs containing 10% R2A following the Poisson distribution, resulting in approximately 8%–12% of GMDs containing a single microbial cell. During incubation, the number of GMDs in which microcolonies formed increased over time in the GMD-agg cultures for both soil- and activated sludge-derived cells. In contrast, little increase was observed in the GMD-dis and DAP cultures, and the colony formation efficiency remained low throughout the incubation period. The mean of the maximum colony formation efficiencies for GMD-agg, GMD-dis, and DAP was 12.3% ± 4.6%, 1.4% ± 0.4%, and 1.4% ± 0.8%, respectively, for soil samples (Fig. 2a), and 24.6% ± 11.5%, 2.8% ± 0.9%, and 1.8% ± 0.9%, respectively, for AS samples (Fig. 2b).

These results indicate that cultivation at high cell densities in GMD-agg promotes the colony formation of environmental microorganisms more effectively than DAP or GMD-dis. The similar performance of GMD-dis and DAP suggests that the increase in colony formation in GMD-agg is not simply due to encapsulation in GMDs or due to a difference in observed colony size between those in GMDs and on plates.

#### Transition of microbial community structure

Next, we examined the microbial community composition to assess how GMD-agg influenced the taxonomic structure using amplicon sequencing of the 16S rRNA V4 region. For community analysis, soil and activated sludge samples were freshly collected and cultured using the GMD-agg and GMD-dis methods, and a tendency toward higher colony formation efficiency was observed under the GMD-agg condition (Fig. S3). In soil samples, community structures differed markedly between GMD-agg and GMD-dis (Fig. 2c; Fig. S4). In GMD-dis, the major taxa, including *Gammaproteobacteria*, *Alphaproteobacteria*, *Bdellovibrionia*, and *Bacteroidia*, were consistent between the early (day 2 and day 5) and late (day 21 and day 28) stages (Fig. 2c). In GMD-agg, however, while similar taxa dominated in the early period, the late-stage communities included additional taxa, such as *Verrucomicrobia*, *Actinomycetia*, *Planctomycetia*, and *Vicinimibacteria*. Notably, *Verrucomicrobia*, *Planctomycetia*, and *Vicinimibacteria* are classes with few known cultured representatives. In contrast, the activated sludge samples showed less pronounced differences between GMD-agg and GMD-dis (Fig. 2d; Fig. S5).

Alpha diversity analysis revealed that diversity indices (Faith’s PD, Shannon entropy, and observed ASVs) were similar between methods on day two but were consistently higher in GMD-agg by day 28 (Fig. 2e and f). Particularly large differences were observed in the observed ASVs. In the activated sludge samples, all diversity measures were also higher in GMD-agg by day 10, although the differences were smaller.

Beta diversity analysis based on Bray-Curtis distances further confirmed these results. Principal coordinate analysis (PCoA) showed distinct separation of the GMD-agg and GMD-dis communities in the soil throughout the incubation period (PERMANOVA: *P* ≤ 0.001) (Fig. S6a). In contrast, no major differences were observed in the AS communities between the two methods (PERMANOVA: *P* = 0.714) (Fig. S6b).

#### Sub-cultivation and isolation

To compare the diversity of sub-cultivable microorganisms obtained by each method, we isolated colonies from GMD-agg, GMD-dis, and DAP cultures. Colonies from GMDs were obtained by plating them on agar and isolating individual colonies. In total, 282 isolates (94 per method) from soil and 240 isolates (80 per method) from activated sludge were randomly selected and identified using 16S rRNA gene sequencing.

From the soil samples, 86 (GMD-agg), 77 (GMD-dis), and 90 (DAP) isolates were successfully identified, representing 21, 14, and 13 OTUs, respectively (Table S1). For activated sludge samples, 69 (GMD-agg), 66 (GMD-dis), and 71 (DAP) isolates were identified, corresponding to 43, 21, and 25 OTUs, respectively (Table S2). The OTU-to-isolate ratios (number of OTUs divided by the total number of isolates) were 0.24 (GMD-agg), 0.18 (GMD-dis), and 0.14 (DAP) for soil (Fig. 2g), and 0.62, 0.32, and 0.35 for activated sludge (Fig. 2h), respectively.

These results indicate that GMD-agg has greater taxonomic diversity among isolates in both sample types.

In both soil and activated sludge, isolates representing OTUs different from those obtained by the agar plate method were recovered using GMD-agg. However, in soil samples, seven OTUs were shared between those derived from GMD-agg and GMD-dis. A binomial model was applied to examine differences in the occurrence of unique OTUs between GMD-agg and GMD-dis, and no significant difference was detected (*P* = 0.487). In activated sludge, many isolates were specific to each method, but a chi-squared test indicated that the difference in the number of isolates obtained was not statistically significant (*P* = 0.2167). Notably, the proportion of potentially novel species (≤97% 16S rRNA similarity to the closest known relative) was higher among GMD-agg isolates in the activated sludge sample (Table S2).

### Rebuilding growth-promoting microbial interactions using isolated strains

To clearly demonstrate that the enhanced colony formation efficiency observed with the GMD-agg method is attributable to microbial interactions among environmental microorganisms, we investigated interactions using isolated strains and the GMD-agg cultivation method. First, we used the GMD-agg method to isolate strains from environmental samples and constructed collections of isolates that would later be used for screening growth-promoting pairs (Fig. S7). In total, 22 strains from the soil sample and 25 strains from the activated sludge sample were collected (Tables S3 and S4).

Most environments are nutrient-limited, and microorganisms inhabiting such environments are frequently exposed to starvation, resulting in many cells entering a low-activity state (36, 37). Consequently, their ability to form colonies can be severely impaired (38). Therefore, we focused on strains that showed low colony formation at the late stationary phase, used here as a starvation condition, specifically those with plating efficiencies of less than 10% on agar plates (Fig. S8). Among the strains with plating efficiency below 10%, we excluded those that did not form any colonies, leaving nine strains from soil and four from activated sludge. Finally, we selected four test strains from soil (G6, G10, G17, and G18) and four from activated sludge (A7, A30, A33, and A38) (Table 1). We then searched for helper strains within the same isolate collection, defined as strains whose supernatants enhance colony formation of the test strains. Cell-free culture supernatants were collected from all isolates at the stationary phase, and agar plates supplemented with 5% (v/v) of each supernatant were prepared. The test strains were inoculated onto these media, and colony-forming units were quantified. We defined as helper strains those isolates whose supernatants yielded particularly high colony formation efficiencies compared with the control medium, which did not contain any supernatant (Table 1; Fig. S9 and S10).

**TABLE 1:** Test strains and each helper strain

Label	Test strain (strain label)	Helper strain (strain label)
Pair 1 from activated sludge	*Vitreoscilla* sp. (A7)	*Acinetobacter* sp. (A37)
Pair 2 from activated sludge	*Duganella* sp. (A30)	*Bosea* sp. (A64)
Pair 3 from activated sludge	*Rhodococcus* sp. (A33)	*Herbaspirillum* sp. (A25)
Pair 4 from activated sludge	*Aquitalea* sp. (A38)	*Bosea* sp. (A64)
Pair 1 from soil	*Bradyrhizobium* sp. (G6)	*Cupriavidus* sp. (G5)
Pair 2 from soil	*Tetrasphaera* sp. (G10)	*Marmoricola* sp. (G13)
Pair 3 from soil	*Flavobacterium* sp. (G17)	–^*a*^
Pair 4 from soil	*Ralstonia* sp. (G18)	*Bacillus* sp. (G02)

^*a*^ No helper strain was identified for strain G17 in this study.

**Fig 3:**
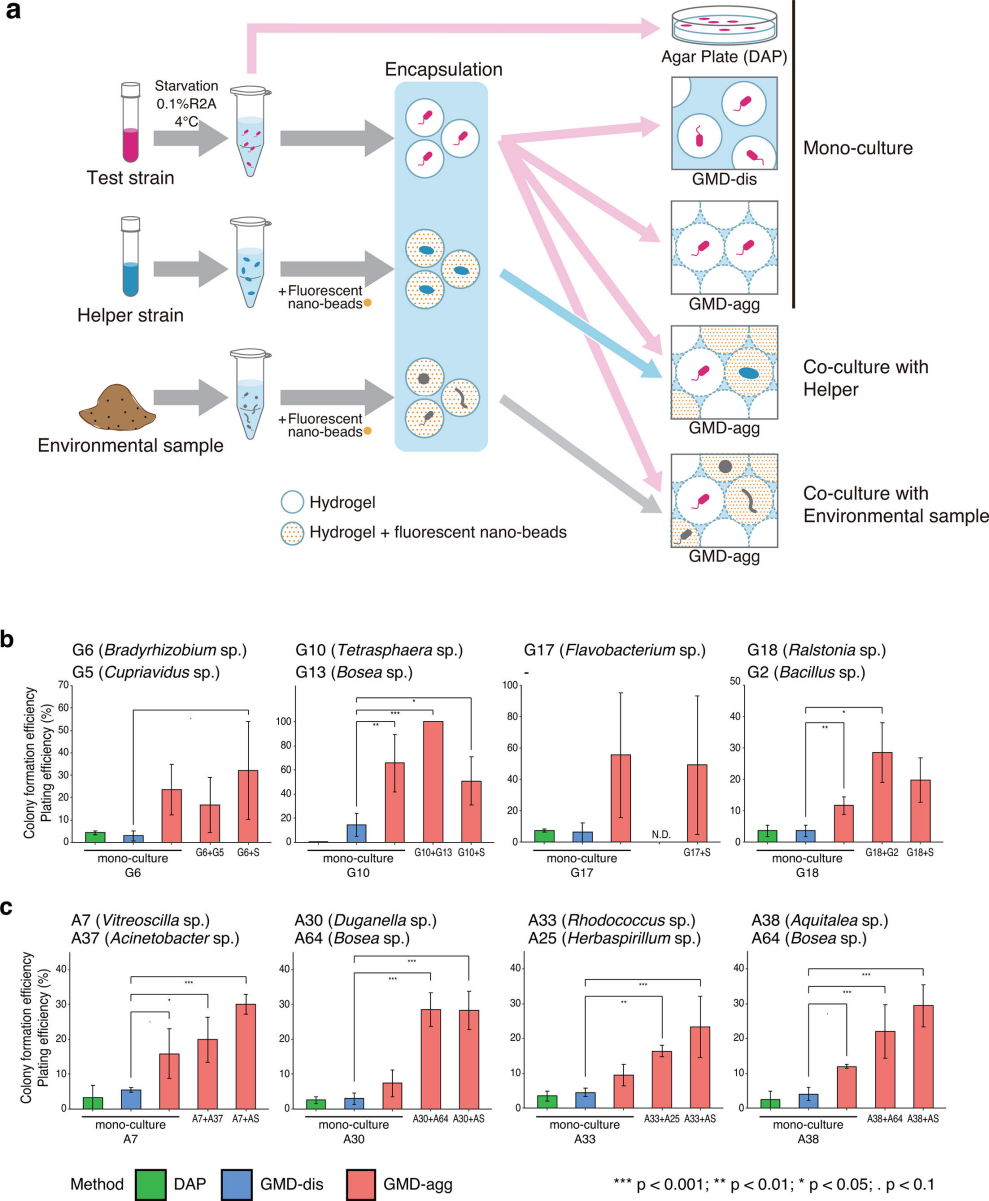
Colony formation efficiencies at the rebuilt growth promoting microbial interactions using isolated strains. (**a**) The test strains were first incubated at 4°C in 0.1% R2A medium to induce a low-activity state. To distinguish the droplets containing the test strains from those containing the helper strain (blue) or environmental microorganisms (gray), the droplets were fluorescently labeled by encapsulating the fluorescent nano-beads. This labeling enabled tracking of test strain growth independently under co-culture conditions. Droplets containing each cell type were prepared separately and used for co-culture in the GMD-agg system or for mono-culture. (**b**) Colony formation efficiencies of test strains isolated from soil (G6, G10, G17, and G18) and (**c**) test strains isolated from activated sludge (A7, A30, A33, and A38) under various culture conditions. “S” indicates soil samples. “AS” indicates activated sludge samples. Error bars represent the standard deviation (SD). Asterisks indicate statistically significant differences compared to GMD-dis and another cultivation method, except for DAP. A one-way ANOVA followed by a Dunnett’s multiple comparisons analysis. N.D., no data.

Next, based on these selected test strains and their helper strains, we designed co-cultivation experiments (Fig. 3a). To evaluate the improvement efficiency from a low-colony-forming state, the test strains were cultured under five different conditions: (i) DAP (mono-culture), (ii) GMD-dis (mono-culture), (iii) GMD-agg (mono-culture), (iv) GMD-agg co-culture with helper strains, and (v) GMD-agg co-culture with environmental microbial community (soil or activated sludge). To distinguish the test strains from helper strains or environmental microbes, GMDs containing the helper strains or environmental microbes were labeled with fluorescent nano-beads, which enabled us to track their growth even under co-culture conditions (Fig. 3a). In addition, to standardize the condition of the test strains, a starvation treatment was applied.

The colony formation efficiency of all test strains was generally low, often below 5%, in DAP and GMD-dis cultures (except for G10 and G17 in GMD-dis). In contrast, GMD-agg co-cultured with helper strains or environmental communities often led to much higher colony formation efficiencies than those observed in pure culture systems (DAP and GMD-dis) (Fig. 3b and c).

For the soil-derived isolates, co-culture of test strains G6, G10, and G18 with their respective helper strains (G5, G13, and G2) in GMD-agg resulted in colony formation efficiencies of 17%, 100%, and 29%, respectively, which was approximately 6.7- and 8.0-fold higher than that of DAP and GMD-dis for G10 + G13 and G18 + G2. Similarly, the co-culture of G6, G10, G17, and G18 with the soil microbial community yielded colony formation efficiencies ranging from 20% to 51%, corresponding to 3.4- to 11-fold increases compared with DAP or GMD-dis (e.g., G6 + S, G10 + S) (Fig. 3b).

For the activated sludge-derived isolates, co-culture of A7, A30, A33, and A38 with their helper strains (A3, A64, A25, and A64) in GMD-agg culture led to colony formation efficiencies that were 3.6-, 9.7-, 3.6-, and 5.4-fold higher, respectively, than in DAP and GMD-dis (e.g., A7 + A3, A30 + A64). Co-culture of these test strains with the activated sludge microbial community also improved colony formation efficiency by 5.5-, 9.3-, 5.1-, and 7.0-fold, respectively (Fig. 3c).

Moreover, even in the absence of helper strains or environmental microbial communities, some test strains (A7, A38, G10, and G18) exhibited 2.9- to 4.4-fold higher colony formation efficiencies under GMD-agg conditions than under DAP or GMD-dis conditions. When comparing mono-culture under the GMD-agg condition with co-culture with the environmental microbial community, no substantial differences were observed for the soil-derived isolates (G6, *P* = 0.45; G10, *P* = 0.38; G17, *P* = 0.58; G18, *P* = 0.13). In contrast, the activated sludge-derived isolates showed significantly higher colony formation efficiencies when co-cultured with the environmental community (A7, *P* = 0.03; A30, *P* < 0.01; A33, *P* = 0.03; A38, *P* < 0.01).

These results indicate that the GMD-agg method can support the growth of microorganisms under difficult-to-culture conditions, most likely by enabling both intra- and inter-species microbial interactions.

## DISCUSSION

The significance of microbial interactions in natural environments remains poorly understood, largely due to technical limitations in assessing microbial growth at the single-cell level under high cell density conditions (>10⁷ cells/mL). In this study, we developed a new cultivation platform, termed the GMD-agg method, in which hydrogel microdroplets (GMDs) encapsulating individual microbial cells are cultured in oil, allowing gentle aggregation. Using this system, we demonstrated that both inter- and intra-species microbial interactions are essential for the growth of diverse microbial taxa, and that such interactions are a major factor contributing to microbial uncultivability. Moreover, we successfully reconstructed growth-promoting interactions using GMD-agg-derived isolates, demonstrating that microbial interactions directly promote microbial growth.

Specifically, the colony formation efficiency in GMD-agg cultures was approximately 10 times higher than that in direct agar plating (DAP). The low colony formation efficiencies observed in DAP (1%–2% for soil and 3%–5% for activated sludge) were consistent with previous studies (1, 2). In contrast, the GMD-dis condition, in which GMDs were physically separated, showed colony formation efficiencies similar to those of the DAP condition, indicating that the enhanced efficiency in GMD-agg is not simply due to droplet encapsulation but rather due to GMD aggregation. In GMD-agg cultures, GMDs remain loosely aggregated, maintaining a spatial distance between cells of approximately 100 μm, thereby allowing an extremely high microbial cell density (10^7^–10^8^ cells/mL) from the start of cultivation. This density approximates that found in soil (31) and is 1,000–10,000 times higher than that in standard agar plating. Under such conditions, water-soluble compounds can diffuse among GMDs, enabling microbial interactions and likely accounting for the higher cultivation efficiency compared to GMD-dis and DAP. Although oxygen accessibility is a potential concern when comparing GMD-agg with DAP, mineral oil is generally known to have high oxygen permeability (39, 40). Given that GMD-dis, which was also cultured in mineral oil, showed colony formation efficiencies similar to those of DAP, oxygen accessibility is likely a minimal factor and not the major contributor to the improved efficiency observed in GMD-agg.

The increase in colony formation observed under high cell density conditions appears inconsistent with previous reports, suggesting an inverse relationship between inoculum size and culturability (41, 42). However, other studies have reported that colony-forming units (CFUs), particularly in the early stages of incubation, can peak at a specific inoculum size (e.g., 3.94 × 10⁴ cells/plate), with lower densities leading to reduced CFUs (43). Thus, the relationship between inoculum size and culturability warrants careful consideration.

GMD-agg also supported the growth of a broader range of microbial taxa than GMD-dis, particularly in soil samples. This suggests that microbial interactions contribute to maintaining high microbial diversity in the soil. While some studies have reported that microbial interactions are predominantly competitive (44, 45), these findings are based on cultured strains and may not reflect the dynamics of natural microbial communities. Our results imply that positive interactions may be more prevalent in diverse uncultured communities. This view is supported by recent studies showing that high community connectivity helps to maintain microbial diversity (11).

In soil samples, completely distinct community structures were observed between GMD-agg and GMD-dis (Fig. 2c). Nevertheless, a substantial number of isolates obtained by GMD-dis were assigned to the same OTUs as those recovered by GMD-agg (Fig. 2g). This likely reflects that the GMD-agg cultivation approach in soil samples still enriches rare taxa that are difficult to isolate. This observation is consistent with the finding that α-diversity after cultivation increased compared to the initial samples.

For soil microbes, it has been reported that colony appearance timing is influenced by lag time and that cells can be grouped into clusters based on this timing (43, 46). If lag time alone determined colony formation, GMD-dis and GMD-agg cultures would yield similar results. However, the distinct community changes observed in GMD-agg suggest that microbial interactions may reduce lag time or induce sequential growth through interaction cascades (47). Thus, microbial interactions appear to support the growth of dormant or slow-growing taxa, enabling the cultivation of a broader range of environmental microbes.

To further examine the role of microbial interactions in growth promotion, we tracked the behavior of specific isolates obtained from GMD-agg cultures. In this experiment, to mimic environmental conditions, we used isolates that had undergone starvation treatment. The response of isolates to starvation varied; however, no relation to phylogeny was observed, indicating that there was no phylogenetic bias in this experiment. When re-incubated into the original environmental microbial community, the colony formation units of the test strains increased (Fig. 3b and c), indicating that growth-promoting interactions occur within natural communities. Although this study tested a limited number of strains, similar effects are likely to be applicable to a broader range of microbes. Colony formation efficiencies were also enhanced when the test strains were co-cultured with helper strains (Fig. 3b and c). Additionally, for some test strains, colony formation efficiency was improved even under mono-culture in GMD-agg, compared with GMD-dis and DAP where microbial interactions are limited. These results suggest that both inter- and intra-species interactions contribute to microbial growth in natural settings. Therefore, initiating cultures at a high cell density, as achieved in GMD-agg, may be critical for cultivating environmental microorganisms, including previously uncultivated taxa. The improvement of colony formation efficiencies under GMD-agg mono-culture conditions was particularly shown in soil-derived strains compared with strains from activated sludge. This finding suggests that the structures of microbial interactions differ between each environment; however, because only a limited number of isolates were tested in this study, further isolation and investigation will be necessary to draw definitive conclusions.

Despite these findings, the specificity of helper strains responsible for inter-species interactions was not fully explored in this study and warrants further investigation. It also remains unclear which substances are responsible for inducing or promoting growth and whether they trigger the initiation of proliferation or accelerate its progression. These aspects will need to be clarified in future studies.

This study introduced two methodological advances that enabled the discovery of previously unrecognized growth-inducing interactions in environmental microbial communities: First, before this work, no method existed that could achieve high cell density (10⁷–10⁸ cells/mL) similar to those in natural environments while maintaining separation between cultures and allowing the exchange of soluble factors. We accomplished this by aggregating the GMDs in oil. During cultivation, individual cultures remained isolated (i.e., pure) but were capable of interacting via diffusible compounds. Second, by encapsulating fluorescent particles within droplets, we were able to identify and track specific cells in co-culture or complex communities. While fluorescent protein labeling is widely used, it is limited to genetically tractable strains. In contrast, our approach allows strain-specific tracking in high-density (10⁷–10⁸ cells/mL) systems that are representative of natural conditions, such as soil. Using this method, we identified various interaction patterns within microbial communities, between species, and even within species that promoted microbial growth.

## MATERIALS AND METHODS

### Environmental sample preparation

Soil samples were collected from a forest area located at Hiroshima University, Higashihiroshima, Hiroshima, Japan. Activated sludge samples were obtained from the wastewater treatment system at the waste treatment plant in Higashihiroshima, Hiroshima, Japan. Soil samples (10 g in 100 mL of 0.1× PBS) and activated sludge samples (20 mL) were homogenized at 30% power for 1 min using an ultrasonicator. The debris in the suspensions was removed by filtration through a 30-μm-pore-size nylon net filter (Merck, USA), a 10-μm-pore-size nylon net filter, and a 5-μm-pore-size cellulose nitrate filter (25-mm diameter; Merck, USA). Microbial cells were concentrated by centrifugation at 20,000 × *g* at room temperature (25°C) for 10 min. For activated sludge, a subsample was additionally filtered at least three times onto a 2-μm-pore-size cellulose nitrate filter (25-mm diameter; Merck, USA) to remove initial microcolonies. Bacterial cells were diluted in buffer (0.1× PBS, pH 7.2). For cell counting, 10 μL of the sample was transferred to a tube, and cells were stained with SYBR Gold. After staining for 5 min, the cells were collected onto a black membrane filter (Merck, USA) and counted under a fluorescence microscope. Following counting, the cell suspension was diluted and adjusted to appropriate cell densities for GMD cultivation and agar plate cultivation.

### Encapsulation into GMD

Microbial cells (100 μL, 10 cellslls/mL) were mixed with 40°C preheated 1.5% low-melting agarose (0.5 mL, Seq plaque, Lonza, USA), 10% (final concentration) R2A (50 μL, Nihon Seiyaku, Japan), and 10% non-ionic surfactant PLURONIC (25 μL, Sigma-Aldrich, USA). The mixture was added to 15 mL of 1% non-ionic surfactant Span 80 (Sigma-Aldrich, USA), including mineral oil (40°C, Sigma-Aldrich, USA) and emulsified at room temperature (25°C) using a CellSys 100 micro drop maker (1,500 rpm, 2 min, One Cell System). The mixture was then cooled with ice under stirring for 7 min at 150 rpm. For GMD-dis cultivation, the GMD-oil mixture was transferred into a vial and incubated under continuous stirring using a magnetic stirrer. For the GMD-agg cultivation system, the following procedure was applied: the GMD-oil mixture was centrifuged at 500 × *g* for 5 min. To remove the surfactant, the pellet was washed twice by centrifugation (500 × *g* for 5 min) with 10% R2A, and the purified GMD pellet containing encapsulated cells was collected. The retrieved GMDs were then suspended in 15 mL of mineral oil without surfactant. The samples were transferred into a bottle and incubated at room temperature (25°C) for 10–28 days, depending on the environmental sample.

For agar plate cultivation, the inoculum was adjusted to contain 5,000 cells per plate and incubated at room temperature under conditions similar to the other cultivation methods.

### Sampling GMDs and observation of microcolonies

For observation, 600-μL emulsions containing GMDs with oil were collected from the culturing bottle into a 1.5-mL tube. To separate GMDs from oil, the emulsion was centrifuged at 500 × *g* for 5 min, and the oil phase was removed. The collected GMDs were washed with 1 mL MilliQ aliquots by centrifugation at 500 × *g* for 5 min in triplicate. The cells or microcolonies in the GMDs were stained with SYBR Green I solution (10^−3^ Molecular Probes, USA) for 5 min in the dark. Subsequently, the number of GMDs, the number of GMDs containing single cells, and the number of GMDs containing microcolonies were counted under a fluorescence microscope. A cluster of four or more adjacent cells was defined as a microcolony. To consider dead cells, the number of GMDs containing observable cells during cultivation; therefore, the initial number of encapsulated cells per GMD was used as a reference, and the colony formation rate on each observation day was normalized by the number of GMDs (Fig. S11). The total number of GMDs was counted based on microscopic images using ImageJ.

The colony formation efficiency of both GMD-agg and GMD-dis was represented as the microcolony number in the GMD to the total encapsulated cell density and calculated using equation 1. Let $G_{0}(t=0)$ denote the count of GMDs at *t* = 0, and $S_{0}(t=0)$ denote the count of GMDs containing single cells at *t =* 0 . Similarly, let $G_{i}(t=i)$ represent the count number of GMDs at time t=i, and $M_{i}(t=i)$ represent the count number of GMDs containing microcolonies at time t=i. Plating efficiency (%) was estimated using equation 2.


(1) $$Colony\;formation\;efficiency\;in\;GMD\;\left(\%\right)=\;\frac{M_{i}}{G_{i}}\times \frac{G_{0}}{S_{0}}\times 100$$

(2) $$Plating\;efficiency\;\left(\%\right)=\;\frac{the\;total\;number\;of\;colonies}{the\;number\;of\;cells\;inoculated\;on\;an\;agar\;plate}\times 100$$

### Community diversity analysis

The GMD cultures intended for community analysis were prepared and cultivated, as previously described. For community analysis, aliquots of 1.5 mL were collected from the cultured samples. To obtain GMDs from these samples, they were washed, and the oil was removed in the same manner as during observation. Microbial DNA was extracted from the GMDs after washing using the DNeasy PowerSoil Pro kit (QIAGEN, Netherlands) according to the manufacturer’s instructions and then stored at −28°C. Community diversity was analyzed using amplicon sequencing targeting the V4 region of 16S rRNA.

The DNAs obtained from the soil samples were targeted to the V4 region of the 16S rRNA gene, and DNAs were amplified with a primer set 515F/806R and enzyme KOD -Plus- Neo (TOYOBO, Japan). The forward primer (5′-ACACTCTTTCCCTACACGACGCTCTTCCGATCTNNNNNGTGCCAGCMGCCGCGGTAA-3′) contained Illumina’s adapter, 0–5 bp error-correcting barcode (designated by NNNNN), and the primer 515F. The reverse primer (5′-GTGACTGGAGTTCAGACGTGTGCTCTTCCGATCTNNNNNGGACTACHVGGGTWTCTAAT-3′) contained Illumina’s adapter, 0–5 bp error-correcting barcode (designated by NNNNN), and the primer 806R. Samples were initially denatured at 94°C for 2 min, then amplified using 30 cycles of 98°C for 10 s, 55°C for 30 s, and 68°C for 20 s. A final extension step at 68°C for 7 min was added at the end of the program to ensure complete amplification of the target region. The first PCR products were purified using VAHTS DNA Clean Beads (Vazyme, China). After purification, these samples were amplified with KOD FX Neo, and sample-specific barcodes were added (2nd PCR). The second PCR was performed for 10 cycles under the following conditions: pre-denaturation at 94°C for 2 min; followed by denaturation at 98°C for 10 s, annealing at 60°C for 30 s, and extension at 68°C for 30 s, with a final extension at 68°C for 2 min. The second PCR products were purified using VAHTS DNA Clean Beads (Vazyme, China), and their quality was confirmed using a Fragment Analyzer with the dsDNA 915 Reagent Kit (Agilent Technologies, USA). Pair-end sequencing was performed on the Illumina MiSeq platform at the Bioengineering Lab. Co., Ltd.

The DNAs obtained from activated sludge samples were targeted to the V4 region of 16S rRNA, and DNAs were amplified with a primer set and KOD -Plus- Neo (TOYOBO, Japan). The forward primer (5′-CCATCTCATCCCTGCGTGTCTCCGACTCAGNNNNNNNNNNNNGTGCCAGCMGCCGCGGTAA-3′) contained Ion PGM’s adapter A, 10 bp barcode for sample index (designated by NNNNN), 2 bp linker, and the primer 515F. The reverse primer (5′-CCTCTCTATGGGCAGTCGGTGATGGACTACHVGGGTWTCTAAT-3′) contained Ion PGM’s adapter P1, and the primer 806R. PCR was performed for 30 cycles under the following conditions: pre-denaturation at 94°C for 2 min; followed by denaturation at 98°C for 10 s, annealing at 60°C for 30 s, and extension at 68°C for 30 s; with a final extension at 68°C for 2 min. The PCR products were purified using AMPure XP beads (Beckman Coulter, USA). DNA concentrations were measured with a Qubit using the dsDNA HS Assay Kit (Thermo Fisher, USA). Subsequently, equal concentrations of amplified DNA from each sample were pooled, and the mixed DNA of the desired length was recovered and purified by gel extraction. Quality was confirmed with a 2100 Bioanalyzer (Agilent Technologies, USA) using the High Sensitivity DNA Kit (Agilent Technologies, USA). Single-end sequencing was performed on the Ion PGM (Thermo Fisher, USA) platform.

Raw reads of 16S rRNA amplicon sequencing were processed according to the QIIME2 pipeline. Quality filtering, paired-end read merging, and chimera checking were performed using DADA2. Sequence classification was based on the Greengenes2 2024.09 database (48). Bar plots for visualization were generated using the phyloseq (v1.42.0) (49) and ggplot2 packages in R. Alpha diversity for samples from the GMD-agg and GMD-dis groups was calculated using the diversity plugin in QIIME two at a sampling depth of 6,000. Beta-diversity analysis was calculated based on the Bray-Curtis distance using the phyloseq package in R, and visualized using principal coordinates analysis (PCoA) plots.

### Sub-culturing from the GMD cultivations

GMDs were collected after the cultivation according to the method described above, followed by oil removal and washing. At this point, each GMD contained at most several hundred cells. Collected GMDs were added to Percoll PLUS/Percoll (0.45 mL, GE Healthcare, USA), 50% non-ionic surfactant Tween 40 (2 μL, Nacalai Tesque, Japan), and 0.15 M NaCl (1 mL, Nacalai Tesque, Japan) and centrifuged at 20,000 × *g* for 1 h. Pure GMDs were retrieved and washed with 1 mL Milli-Q aliquots by centrifugation at 10,000 rpm for 5 min. To inoculate without disrupting the GMD structure and while preserving the colonies, approximately 30,000 GMDs were added to 10 mL of tempered (40°C) 1% low-melting agarose (Sea Plaque, Lonza, USA), mixed, poured onto each 10% R2A agar plate by dividing into three equal amounts, and incubated at room temperature. After incubation, 100 colonies were randomly selected and subcultured onto agar plates.

### Identification of isolates by 16S rRNA sequencing

After incubation, 80–100 colonies were randomly selected from each cultivation method. The 16S rRNA of isolates was amplified by colony direct PCR. We amplified the 16S rRNA gene using a 27F/1492R primer set (50) and KOD FX Neo (TOYOBO, Japan). PCR solutions were initially denatured at 94°C for 2 min, then amplified by using 30 cycles of 98°C for 10 s, 55°C for 30 s, and 68°C for 20 s. A final extension of 7 min at 68°C was added at the end of the program to ensure complete amplification of the target region. The PCR products were sequenced with the primer 805R (5′-GACTACCAGGGTATCTAATC-3′) (51) at Takara Bio Inc. (Japan). Considered clones, as sequences longer than 500 nucleotides (nt) and non-chimeric, were estimated with databases in GenBank (https://www.ncbi.nlm.nih.gov/genbank/) (52, 53) to identify the closest matching sequences using BLASTn (54). The chi-squared test and the binomial model were performed using the stats package in R.

### Selection of test and helper strains for co-cultivation experiments

The isolates derived from GMD-agg in AS used in this study were obtained from the strains listed in Table S2, whereas the isolates derived from GMD-agg in soil samples were newly acquired. All isolates used in the experiments are shown in Tables S3 and S4.

Isolates that treated starvation were cultured on agar plates, and cells exhibiting low plating efficiency were selected as test strains. Each isolate was first grown in 10% R2A broth until reaching the late stationary phase. Cells were then harvested by centrifugation at 14,000 ×*g* for 10 min, washed twice with 0.1× PBS, and resuspended in 0.1× PBS. A portion of the cell suspension was stained with SYBR Gold and counted using fluorescence microscopy to adjust the inoculum concentration. Each strain was inoculated with 500 cells onto a 10% R2A agar plate and incubated at room temperature for 1 week. Plating efficiency was calculated as the ratio of the number of colonies to the inoculated 500 cells. From strains exhibiting sufficiently low plating efficiency (0.5%–10%), eight strains (four from soil and four from AS) were selected as test strains (Fig. S8).

For the selection of helper strains, test strains were cultured in 10% R2A broth, centrifuged at 14,000 × *g* for 10 min, and washed twice with 0.1× PBS. The washed pellet was resuspended in 0.1% R2A broth and incubated at 4°C for 3 days to induce starvation. After starvation, the suspension was centrifuged again at 14,000 × g for 10 min, the supernatant was removed, and the pellet was resuspended in 0.1× PBS. To prepare agar plates containing supernatants from each isolate, all isolates were cultured in 10% R2A broth, and the culture supernatants were collected. The supernatants were then autoclaved. Finally, 10% R2A agar plates containing each culture supernatant (final concentration 1%) were prepared. The 500 cells of test strains after starvation were inoculated onto agar plates with the modified 10% R2A (including supernatants) and the plate with standard 10% R2A (without supernatant). After 1 week of incubation, colony numbers were counted and compared between the standard and modified plates (Fig. S9 and S10). Finally, one helper strain was selected for each strain from the activated sludge, and 0 or 1 for each strain from the soil. The helper strains were used for the following co-cultivation test.

### Tracking the colony formation of the test strain in co-culturing oil cultivation

For the GMD-based co-culture experiments, GMDs encapsulating each sample were prepared under each cultivation condition. All test strains were pre-cultured in 10% R2A broth and then incubated at 4°C for 3 days to induce starvation. To distinguish GMDs containing test strains from those containing other microorganisms, fluorescent nanobeads (Polyscience, Inc., USA) were used for labeling. During GMD preparation, fluorescent nanobeads were mixed with the microbial cell suspension and gelling agent, resulting in approximately 10–30 beads per GMD. For co-culture with a specific helper strain, test strain cells were encapsulated in non-fluorescent-labeled GMDs, whereas the helper strain cells were encapsulated in fluorescently labeled GMDs containing fluorescent nanobeads. After separately preparing the two types of GMDs, the GMDs were mixed and co-cultured in oil for 240 h. In parallel, the growth of the test strains was assessed under two additional conditions: GMD-dis culture and standard agar plates. Colony formation efficiency and plating efficiency under each condition were calculated, as previously described. Differences in the colony formation efficiency of test strains among each condition were analyzed using one-way ANOVA followed by Dunnett’s test for multiple comparisons, as implemented in the multcomp package in R.

## Data Availability

Amplicon sequence data were deposited to NCBI under the BioProject accession number PRJDB20252. For isolated strains, 16S rRNA gene sequences were deposited to GenBank under the accession numbers PQ128860–PQ128907, ON680767–ON680791, ON819649–ON819718, PP716552–PP716569, and PP843538.

## SUPPLEMENTAL MATERIAL

Supplemental material mbio.03953-25-s0001.docx Supplemental methods, Figures S1–S11, and Tables S1–S4.

ASM does not own the copyrights to Supplemental Material that may be linked to, or accessed through, an article. The authors have granted ASM a non-exclusive, world-wide license to publish the Supplemental Material files. Please contact the corresponding author directly for reuse.
