# Supplementary material for: High-density microdroplet cultivation reveals the essential role of microbial interactions in the growth of environmental microbes
Source: mBio. 2026 Apr 10;17(5):e03953-25. doi: 10.1128/mbio.03953-25 (PMC13170335; doi:10.1128/mbio.03953-25)
Supplement: Supplemental material — Supplemental methods, Figures S1–S11, and Tables S1–S4. [file mbio.03953-25-s0001.docx]

# Supplemental Methods

## Confirmation of spatial separation

GMDs encapsulating *E. coli* were prepared as previously described, including LB medium (25 g/L). Separately, GMDs containing green fluorescent nano-beads (Polyscience, Inc., USA) were also prepared. Equal volumes (500 µL each) of the *E. coli* containing GMDs and the Fluorescent nano-beads containing GMDs were mixed and incubated at room temperature in the GMD-agg method.

Similarly, mixtures of GMDs containing the soil sample and those containing the fluorescent nano-beads were prepared and incubated at room temperature in the GMD-agg method.

The *E. coli* containing GMDs were collected for observation at 0 h, 23 h, 41 h, 112 h, and 375 h after the start of incubation. Cells were stained with propidium iodide (PI) and observed using fluorescence microscopy. At each time point, 1000 fluorescently labeled GMDs were examined to confirm the presence or absence of cells. The soil containing GMDs was similarly collected and observed at 0 h, 66 h, 162 h, and 400 h.

## Confirmation of Compound Diffusion

GMDs encapsulating *E. coli* were prepared as previously described, including LB medium (25 g/L). Separately, GMDs containing sodium azide (NaN₃), a known inhibitor of *E. coli* growth, were prepared at a final concentration of 2.5 g/L. Equal volumes (500 µL each) of the *E. coli* containing GMDs and the sodium azide containing GMDs were mixed and incubated at room temperature in the GMD-agg method. As a control, empty GMDs (without sodium azide) were prepared and mixed in equal volumes (500 µL each) with the *E. coli* containing GMDs, followed by incubation under the same GMD-agg conditions at room temperature. After 20 hours of incubation, samples were collected, stained with SYBR Green, and observed under a fluorescence microscope to observe cells and colony formation.

# Supplemental Figures/Tables

**Fig S1 Evaluation of microbial cell transfer between GMDs in the GMD-agg condition.**

(a) GMDs containing either E. coli or green fluorescent beads were prepared separately and then incubated together for 375 h using the GMD-agg method. After incubation, the cells were stained with a high concentration of propidium iodide (PI) and examined using fluorescence microscopy. (b) Similarly, GMDs encapsulating soil bacteria and GMDs containing green fluorescent nanoparticles were incubated together for 400 h. Red fluorescence indicates bacterial cells, and green fluorescence indicates fluorescent nano-beads. Scale bars: 50 µm.

**Fig S2 Sharing of water-soluble substances among GMDs in the GMD-agg cultivation system.**

(a) GMDs encapsulating E. coli and GMDs containing sodium azide (NaN₃, 2.5 g/L) were prepared separately and incubated together for 20 h using the GMD-agg method. The cells were stained with SYBR Green and examined using fluorescence microscopy. (b) GMDs encapsulating E. coli were prepared and incubated alone under the same conditions for 20 h. The cells were subsequently stained with SYBR Green and examined using fluorescence microscopy. Scale bar: 50 µm.

**
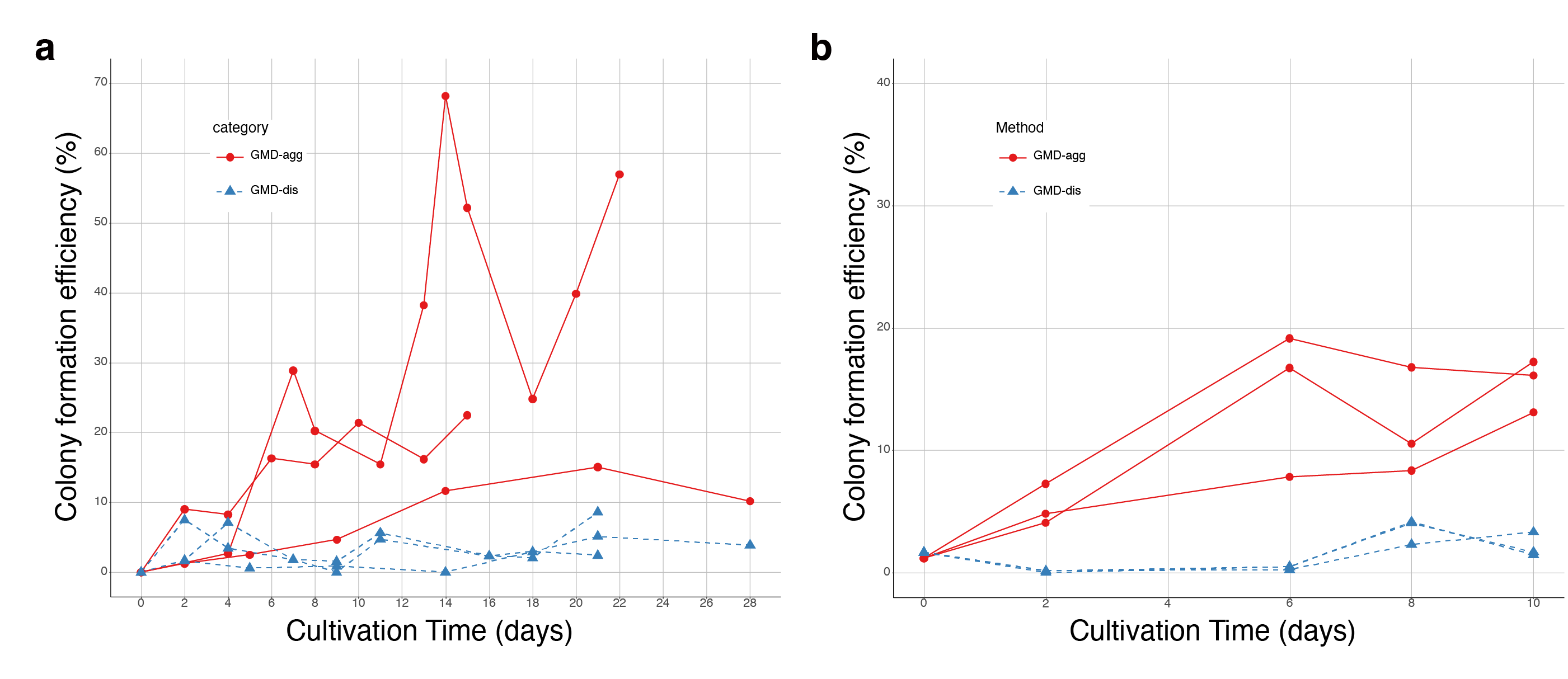
**

**Fig S3 Tracking of colony formation over time for community analysis.**

(a) Temporal changes in the colony formation efficiency within GMDs during GMD-agg and GMD-dis cultivation of soil samples for community analysis. Soil samples were collected from the same site at four different time points and cultured. (b) Temporal changes in the colony formation rate within GMDs during GMD-agg and GMD-dis cultivation of activated sludge samples for community analysis. Activated sludge samples were taken from the same source, and three replicates were prepared and cultured. **Red circles and lines represent GMD aggregation in oil (GMD-agg), blue triangles and dashed lines represent GMD dispersion in oil (GMD-dis).**

**Fig S4 Microbial community dynamics during the cultivation of the soil samples at the phylum level.**

**The results of GMD-agg are shown on the left and those of GMD-dis are shown on the right. ASVs with a relative abundance below 1% were grouped as “Other.” Soil samples were collected from the same site at four different time points and cultured.**

**Fig S5 Microbial community dynamics during the cultivation of the activated sludge samples at the phylum level.**

**The results of GMD-agg are shown on the left and those of GMD-dis are shown on the right. ASVs with a relative abundance below 1% were grouped as “Other.” Activated sludge samples were taken from the same source, and three replicates were prepared and cultured.**

**Fig S6 β-diversity of GMDs and inoculated source samples from soil (a) and activated sludge (b).**

β-diversity was assessed using Bray–Curtis dissimilarities and visualized by principal coordinates analysis (PCoA). Dot shapes represent the sample source or cultivation method, while colors indicate cultivation time. Ellipses denote 95% confidence intervals for each group. Soil samples were collected from the same site at four different time points and cultured. Activated sludge samples were taken from the same source, and three replicates were prepared and cultured.

**Fig S7 Overview of the selection test for the test strains and helper strains**

Details are provided in the Methods section of the main text and in the subsequent figures.


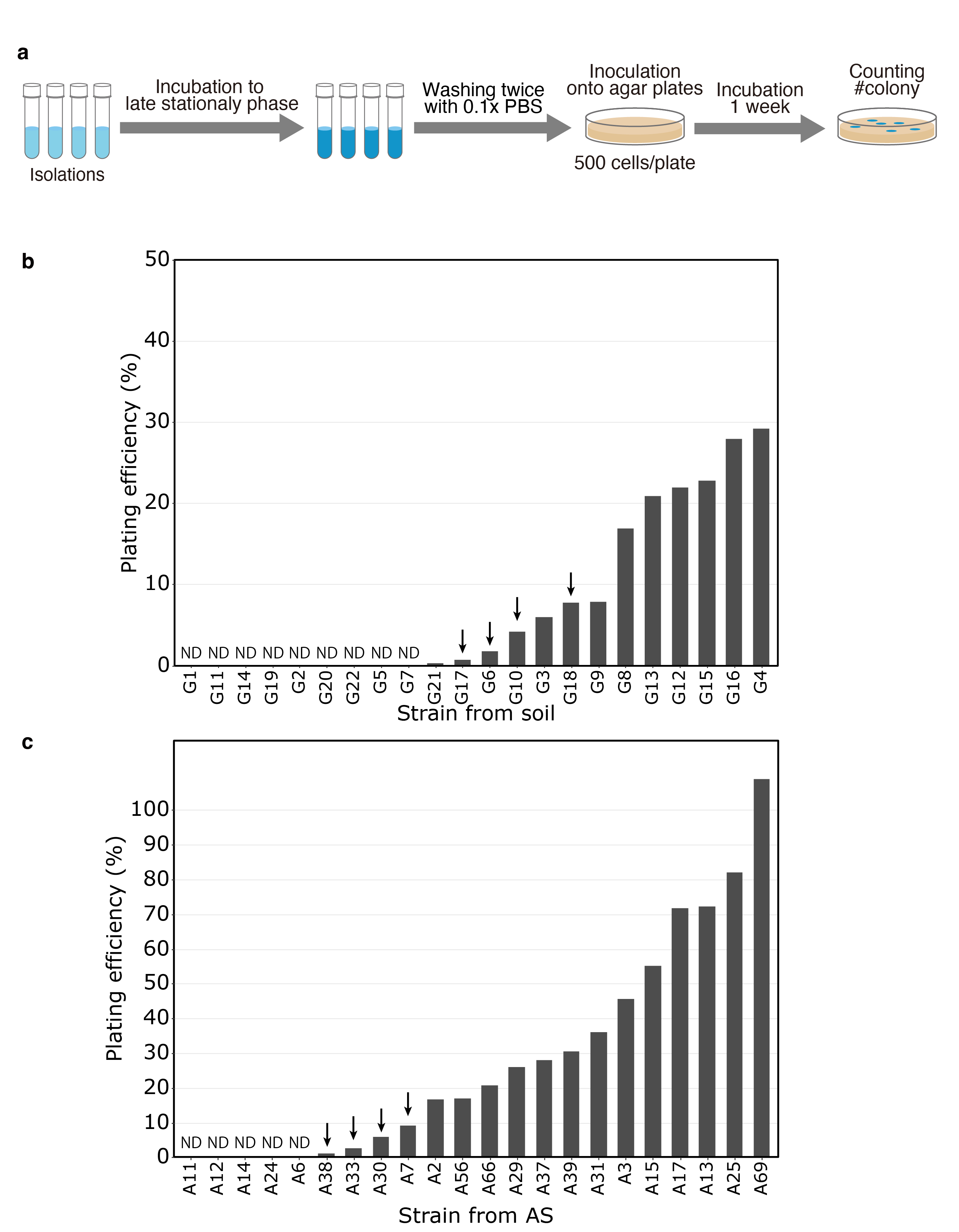


**Fig S8 Plating efficiency of isolates collected from the late stationary phase.**

(a) Overview of the experiment. Each isolate was cultured until the late stationary phase, washed with 0.1× PBS, and inoculated onto agar plates at 500 cells per plate. After one week of incubation, colonies were counted, and plating efficiency was calculated based on the number of inoculated cells (500 cells). (b) Plating efficiency of soil-derived isolates (Table S3). (c) Plating efficiency of activated sludge-derived isolates (Table S4). Arrows indicate selected strains as test strain. Experiments were conducted in triplicate. ND indicates not detected (no colony).


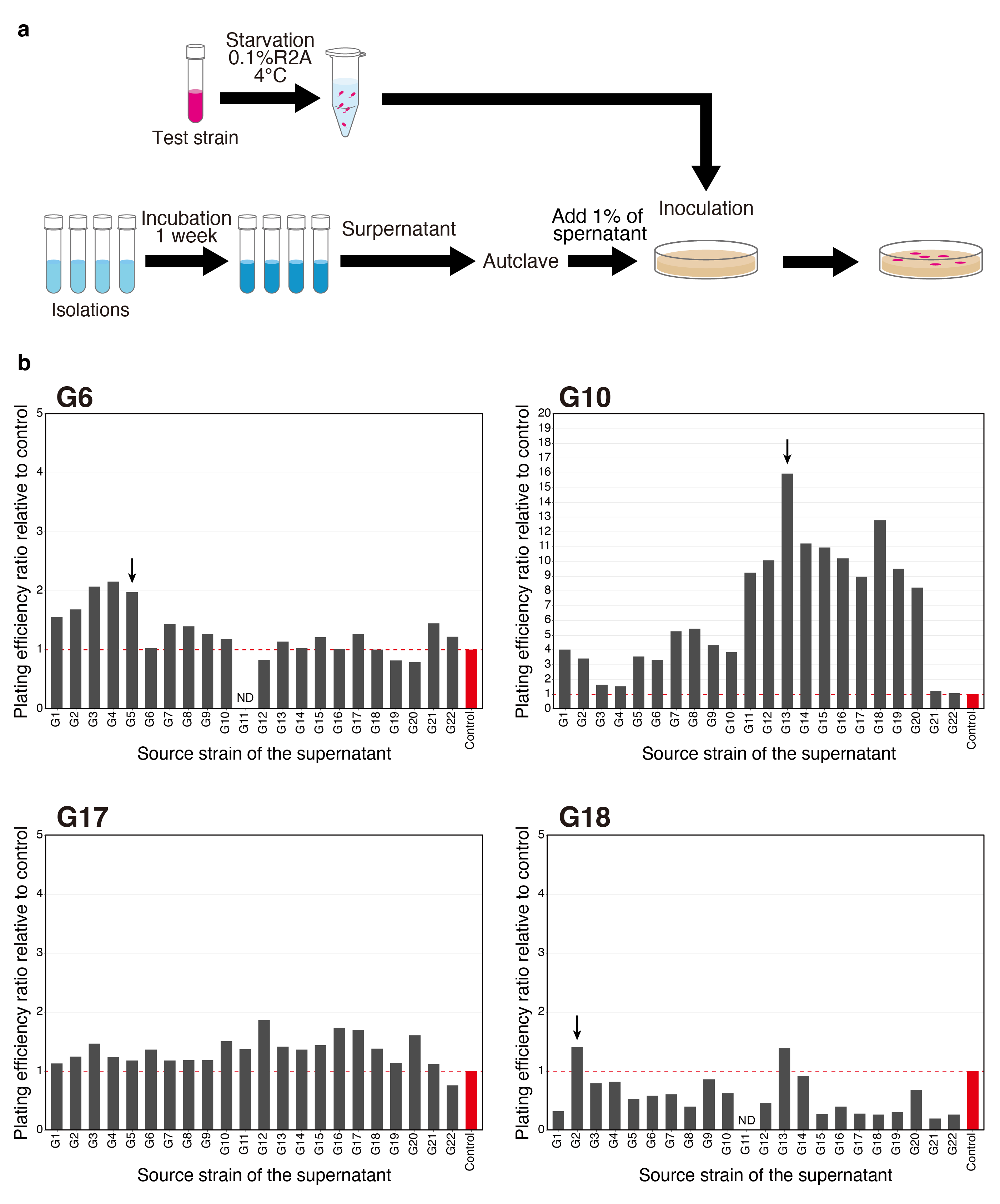


**Fig S9 Plating efficiency of test strain in response to supernatants from soil derived isolates.**

(a) Overview of the experiment. Starved test strains were inoculated onto agar plates containing autoclaved supernatants from individual isolates, and colony numbers were counted after incubation. (b) Response of test strains (G6, G10, G17, G18) to the supernatants of each isolate. The ratio of colony numbers formed on agar plates containing supernatants to those on plates without supernatants is shown. Plates without supernatants are indicated as “Control” (red bars). Arrows indicate helper strains used in following experiments for each test strain. For G17, no helper strain was selected because the difference in colony numbers between plates containing supernatant from G17 and other conditions was minimal. Experiments were conducted in triplicate. ND indicates not detected.


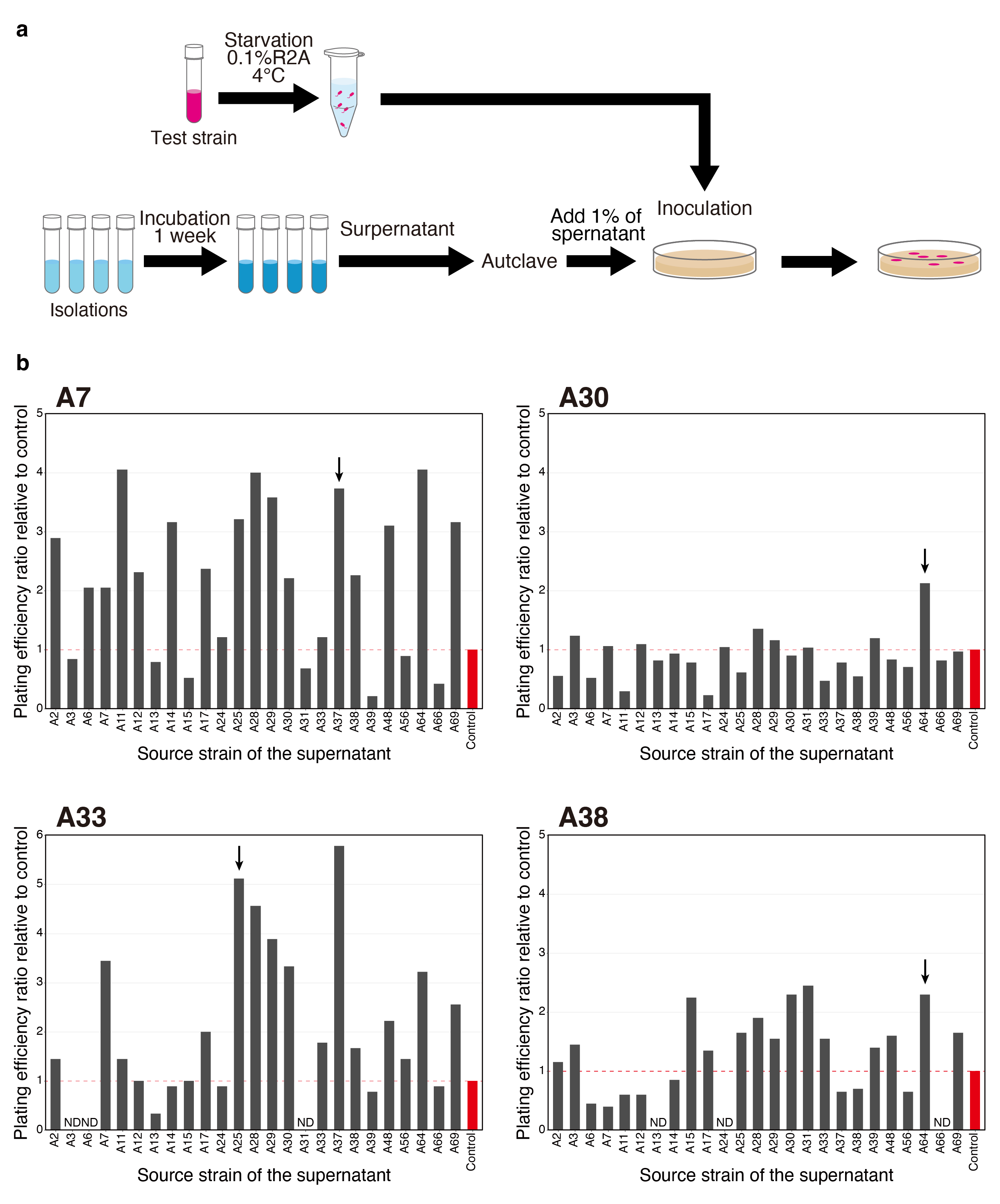


**Fig S10 Plating efficiency of test strain candidates from activated sludge.**

(a) Overview of the experiment. Starved test strains were inoculated onto agar plates containing autoclaved supernatants from individual isolates, and colony numbers were counted after incubation. (b) Response of test strains (A7, A30, A33, A38) to the supernatants of each isolate. The ratio of colony numbers formed on agar plates containing supernatants to those on plates without supernatants is shown. Plates without supernatants are indicated as “Control” (red bars). Arrows indicate helper strains used in following experiments for each test strain. Experiments were conducted in triplicate. ND indicates not detected.

**
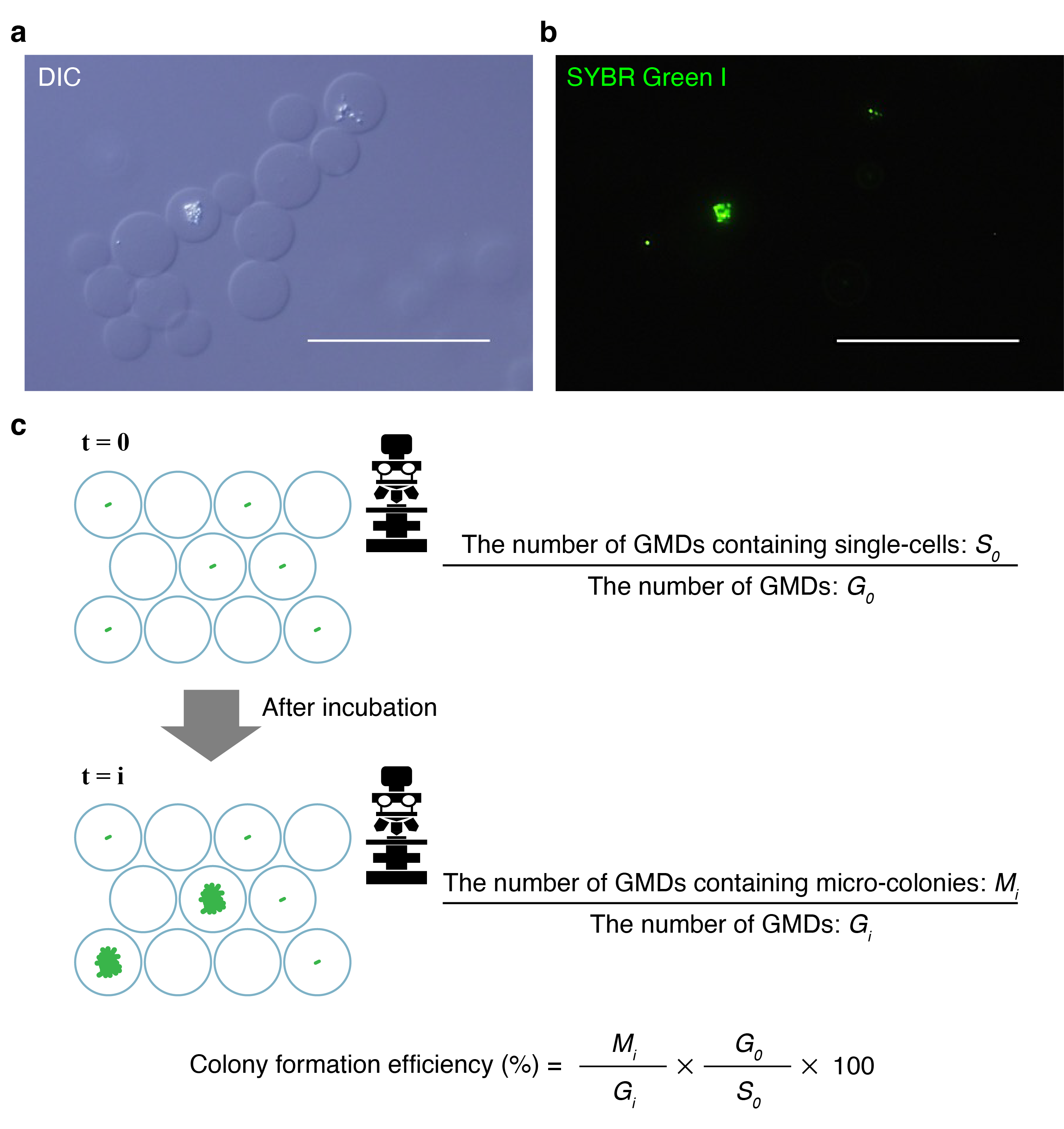
**

**Fig S11 Observation of colonies within GMDs and an overview of the method for calculating colony formation rate.**

(a) Differential interference contrast (DIC) images of GMDs after cultivation. (b) Fluorescence microscopy images of GMDs stained with SYBR Green I after cultivation. (c) Schematic diagram illustrating the calculation of the colony formation efficiency (%) in GMD cultures. Scale bars represent 100 μm.

**Table S1** **Isolates obtained from soil through each method (GMD-agg, GMD-dis, DAP)**

| **Soil GMD-agg** |  |  |  |  |
| --- | --- | --- | --- | --- |
| **Taxonomic group** | **Closest species** | **Similarity (%)** | **No.**^†^ | **Accession no.** |
| *Actinomycetota* | *Microbacterium paraoxydans* | 99.8 | 18 | PQ128874 |
| *Actinomycetota* | *Microbacterium liquefaciens* | 99.8 | 5 | PQ128875 |
| *Actinomycetota* | *Microbacterium lacus* | 99.0 | 1 | PQ128876 |
| *Actinomycetota* | *Microbacterium dextranolyticum* | 97.5 | 1 | PQ128877 |
| *Actinomycetota* | *Agromyces arachidis* | 98.2 | 3 | PQ128878 |
| *Actinomycetota* | *Oerskovia paurometabola* | 100.0 | 6 | PQ128879 |
| *Actinomycetota* | *Cellulosimicrobium funkei* | 99.8 | 5 | PQ128880 |
| *Bacteroidota* | *Chryseobacterium lactis* | 99.0 | 2 | PQ128860 |
| *Alphaproteobacteria* | *Brevundimonas vesicularis* | 100.0 | 8 | PQ128867 |
| *Alphaproteobacteria* | *Bosea lupini* | 99.8 | 2 | PQ128868 |
| *Alphaproteobacteria* | *Agrobacterium larrymoorei* | 100.0 | 2 | PQ128869 |
| *Alphaproteobacteria* | *Pararhizobium arenae* | 99.8 | 1 | PQ128870 |
| *Alphaproteobacteria* | *Brucella pseudogrignonensis* | 100.0 | 1 | PQ128871 |
| *Alphaproteobacteria* | *Novosphingobium lindaniclasticum* | 99.2 | 3 | PQ128872 |
| *Alphaproteobacteria* | *Sphingobium yanoikuyae* | 99.7 | 1 | PQ128873 |
| *Gammaproteobacteria* | *Pseudocitrobacter faecalis* | 100.0 | 4 | PQ128861 |
| *Gammaproteobacteria* | *Citrobacter cronae* | 99.7 | 1 | PQ128862 |
| *Gammaproteobacteria* | *Erwinia rhapontici* | 98.1 | 7 | PQ128863 |
| *Gammaproteobacteria* | *Enterobacter sichuanensis* | 100.0 | 2 | PQ128864 |
| *Gammaproteobacteria* | *Pseudomonas alloputida* | 99.7 | 3 | PQ128865 |
| *Gammaproteobacteria* | *Pseudomonas rhizoryzae* | 99.3 | 10 | PQ128866 |
|  |  |  |  |  |
| Species no. | 21 |  |  |  |
| Isolates no. | 86 |  |  |  |
| Species no. / Isolates no. | 0.24 |  |  |  |

| **Soil GMD-dis** |  |  |  |  |
| --- | --- | --- | --- | --- |
| **Taxonomic group** | **Closest species** | **Similarity (%)** | **No.**^†^ | **Accession no.** |
| *Actinomycetota* | *Microbacterium paraoxydans* | 99.8 | 8 | PQ128881 |
| *Actinomycetota* | *Microbacterium liquefaciens* | 100.0 | 5 | PQ128882 |
| *Alphaproteobacteria* | *Sphingobium yanoikuyae* | 97.8 | 16 | PQ128883 |
| *Alphaproteobacteria* | *Agrobacterium larrymoorei* | 99.7 | 11 | PQ128884 |
| *Alphaproteobacteria* | *Pararhizobium arenae* | 100.0 | 1 | PQ128885 |
| *Gammaproteobacteria* | *Pseudomonas entomophila* | 100.0 | 22 | PQ128886 |
| *Gammaproteobacteria* | *Pseudomonas nicosulfuronedens* | 100.0 | 3 | PQ128887 |
| *Gammaproteobacteria* | *Stenotrophomonas panacihumi* | 100.0 | 1 | PQ128888 |
| *Gammaproteobacteria* | *Stenotrophomonas nematodicola* | 99.8 | 2 | PQ128889 |
| *Gammaproteobacteria* | *Citrobacter cronae* | 99.7 | 1 | PQ128890 |
| *Gammaproteobacteria* | *Enterobacter chuandaensis* | 99.5 | 1 | PQ128891 |
| *Gammaproteobacteria* | *Enterobacter sichuanensis* | 99.7 | 4 | PQ128892 |
| *Gammaproteobacteria* | *Citrobacter telaviensis* | 99.7 | 1 | PQ128893 |
| *Gammaproteobacteria* | *Escherichia marmotae* | 99.8 | 1 | PQ128894 |
|  |  |  |  |  |
| Species no. | 14 |  |  |  |
| Isolates no. | 77 |  |  |  |
| Species no. / Isolates no. | 0.18 |  |  |  |

| **Soil DAP** |  |  |  |  |
| --- | --- | --- | --- | --- |
| **Taxonomic group** | **Closest species** | **Similarity (%)** | **No.**^†^ | **Accession no.** |
| *Actinomycetota* | *Amnibacterium flavum* | 98.3 | 1 | PQ128895 |
| *Actinomycetota* | *Oerskovia paurometabola* | 100.0 | 1 | PQ128896 |
| *Actinomycetota* | *Micromonospora ovatispora* | 100.0 | 3 | PQ128897 |
| *Actinomycetota* | *Micromonospora spongicola* | 100.0 | 1 | PQ128898 |
| *Alphaproteobacteria* | *Bradyrhizobium centrosematis* | 100.0 | 68 | PQ128902 |
| *Alphaproteobacteria* | *Brevundimonas bullata* | 99.2 | 1 | PQ128903 |
| *Alphaproteobacteria* | *Mesorhizobium terrae* | 100.0 | 2 | PQ128904 |
| *Alphaproteobacteria* | *Sediminibacterium magnilacihabitans* | 99.7 | 2 | PQ128905 |
| *Alphaproteobacteria* | *Chryseobacterium mulctrae* | 98.7 | 5 | PQ128906 |
| *Alphaproteobacteria* | *Neoroseomonas lacus* | 100.0 | 1 | PQ128907 |
| *Gammaproteobacteria* | *Pelomonas aquatica* | 99.3 | 3 | PQ128899 |
| *Gammaproteobacteria* | *Varivorax dokdonensis* | 98.5 | 1 | PQ128900 |
| *Gammaproteobacteria* | *Acinetobacter beijerinckii* | 99.7 | 1 | PQ128901 |
|  |  |  |  |  |
| Species no. | 13 |  |  |  |
| Isolates no. | 90 |  |  |  |
| Species no. / Isolates no. | 0.14 |  |  |  |

^†^The number of isolated colonies

**Table S2 Isolates obtained from AS through each method (GMD-agg, GMD-dis, DAP)**

| **AS GMD-agg** |  |  |  |  |
| --- | --- | --- | --- | --- |
| **Taxonomic group** | **Closest species** | **Similarity (%)** | **No.**^†^ | **Accession no.** |
| *Actinobacteria* | *Microbacterium hominis* | 99 | 4 | ON819649 |
| *Actinobacteria* | *Microbacterium lacus* | 100 | 1 | ON819650 |
| *Actinobacteria* | *Microbacterium maritypicum* | 100 | 2 | ON819651 |
| *Actinobacteria* | *Microbacterium oxydans* | 100 | 2 | ON819652 |
| *Actinobacteria* | *Rhodococcus qingshengii* | 100 | 1 | ON819653 |
| *Bacteroidetes* | *Chryseobacterium scophthalmum* | 99 | 1 | ON819654 |
| *Bacteroidetes* | *Cloacibacterium caeni* | 100 | 5 | ON819655 |
| *Bacteroidetes* | *Empedobacter falsenii* | 100 | 1 | ON819656 |
| *Firmicutes* | *Lactococcus chungangensis* | 100 | 2 | ON819657 |
| *Alphaproteobacteria* | *Allorhizobium borbori* | 100 | 2 | ON819658 |
| *Alphaproteobacteria* | *Aquidulcibacter paucihalophilus* | 92 | 1 | ON819659 |
| *Alphaproteobacteria* | *Bosea lupini* | 99 | 1 | ON819660 |
| *Alphaproteobacteria* | *Brevundimonas albigilva* | 96 | 1 | ON819661 |
| *Alphaproteobacteria* | *Brevundimonas terrae* | 99 | 1 | ON819662 |
| *Alphaproteobacteria* | *Novosphingobium aromaticivorans* | 98 | 2 | ON819663 |
| *Alphaproteobacteria* | *Novosphingobium lentum* | 98 | 1 | ON819664 |
| *Alphaproteobacteria* | *Novosphingobium lentum* | 94 | 1 | ON819665 |
| *Alphaproteobacteria* | *Novosphingobium meiothermophilum* | 97 | 2 | ON819666 |
| *Alphaproteobacteria* | *Rhizobium pakistanense* | 98 | 2 | ON819667 |
| *Alphaproteobacteria* | *Rhodopseudomonas telluris* | 99 | 3 | ON819668 |
| *Alphaproteobacteria* | *Sphingomonas aquatilis* | 100 | 1 | ON819669 |
| *Betaproteobacteria* | *Acidovorax wautersii* | 100 | 1 | ON819670 |
| *Betaproteobacteria* | *Aquitalea pelogenes* | 100 | 2 | OP020674 |
| *Betaproteobacteria* | *Duganella rivi* | 100 | 1 | ON819671 |
| *Betaproteobacteria* | *Herbaspirillum aquaticum* | 100 | 1 | ON819672 |
| *Betaproteobacteria* | *Ottowia beijingensis* | 98 | 1 | ON819673 |
| *Betaproteobacteria* | *Pelomonas aquatica* | 99 | 3 | ON819674 |
| *Betaproteobacteria* | *Caldimonas brevitalea* | 98 | 4 | ON819675 |
| *Betaproteobacteria* | *Vitreoscilla stercoraria* | 96 | 1 | ON819676 |
| *Betaproteobacteria* | *Vogesella indigofera* | 92 | 1 | ON819677 |
| *Betaproteobacteria* | *Zoogloea caeni* | 97 | 1 | ON819678 |
| *Gammaproteobacteria* | *Acinetobacter chengduensis* | 100 | 1 | ON819679 |
| *Gammaproteobacteria* | *Acinetobacter guillouiae* | 98 | 2 | ON819680 |
| *Gammaproteobacteria* | *Acinetobacter johnsonii* | 99 | 2 | ON819681 |
| *Gammaproteobacteria* | *Acinetobacter junii* | 99 | 1 | ON819682 |
| *Gammaproteobacteria* | *Acinetobacter parvus* | 98 | 1 | ON819683 |
| *Gammaproteobacteria* | *Acinetobacter seifertii* | 100 | 1 | ON819684 |
| *Gammaproteobacteria* | *Acinetobacter tandoii* | 99 | 1 | ON819685 |
| *Gammaproteobacteria* | *Acinetobacter vivianii* | 99-100 | 2 | ON819686 |
| *Gammaproteobacteria* | *Aeromonas caviae* | 100 | 2 | ON819687 |
| *Gammaproteobacteria* | *Stenotrophomonas terrae* | 99 | 1 | ON819688 |
| *Gammaproteobacteria* | *Thermomonas carbonis* | 98 | 1 | ON819689 |
| *Gammaproteobacteria* | *Thermomonas fusca* | 96 | 1 | ON819690 |
|  |  |  |  |  |
| Species no. | 43 |  |  |  |
| Isolates no. | 69 |  |  |  |
| Species no. / Isolates no. | 0.62 |  |  |  |

| **AS GMD-dis** |  |  |  |  |
| --- | --- | --- | --- | --- |
| **Taxonomic group** | **Closest species** | **Similarity (%)** | **No.**^†^ | **Accession no.** |
| *Actinobacteria* | *Microbacterium flavum* | 98 | 1 | ON819698 |
| *Flavobacteria* | *Chryseobacterium rhizoplanae* | 96 | 1 | ON819699 |
| *Flavobacteria* | *Chryseobacterium rhizoplanae* | 99 | 8 | ON819700 |
| *Flavobacteria* | *Chryseobacterium sediminis* | 99 | 10 | ON819701 |
| *Alphaproteobacteria* | *Agrobacterium radiobacter* | 100 | 1 | ON819702 |
| *Alphaproteobacteria* | *Allorhizobium borbori* | 100 | 10 | ON819703 |
| *Alphaproteobacteria* | *Novosphingobium mathurense* | 99 | 1 | ON819704 |
| *Alphaproteobacteria* | *Ochrobactrum pituitosum* | 100 | 1 | ON819705 |
| *Betaproteobacteria* | *Comamonas koreensis* | 98 | 1 | ON819706 |
| *Betaproteobacteria* | *Pseudoduganella danionis* | 99 | 1 | ON819707 |
| *Gammaproteobacteria* | *Acinetobacter baumannii* | 99 | 1 | ON819708 |
| *Gammaproteobacteria* | *Acinetobacter johnsonii-a* | 96 | 1 | ON819709 |
| *Gammaproteobacteria* | *Acinetobacter johnsonii* | 98 | 4 | ON819710 |
| *Gammaproteobacteria* | *Enterobacter cancerogenus* | 100 | 8 | ON819711 |
| *Gammaproteobacteria* | *Enterobacter cloacae* | 100 | 1 | ON819712 |
| *Gammaproteobacteria* | *Enterobacter roggenkampii* | 99 | 1 | ON819713 |
| *Gammaproteobacteria* | *Pseudomonas mosselii* | 100 | 8 | ON819714 |
| *Gammaproteobacteria* | *Serratia liquefaciens* | 100 | 1 | ON819715 |
| *Gammaproteobacteria* | *Serratia marcescens* | 99 | 2 | ON819716 |
| *Gammaproteobacteria* | *Stenotrophomonas lactitubi* | 99 | 2 | ON819717 |
| *Gammaproteobacteria* | *Stenotrophomonas terrae* | 99 | 2 | ON819718 |
|  |  |  |  |  |
| Species no. | 21 |  |  |  |
| Isolates no. | 66 |  |  |  |
| Species no. / Isolates no. | 0.32 |  |  |  |

| **AS DAP** |  |  |  |  |
| --- | --- | --- | --- | --- |
| **Taxonomic group** | **Closest species** | **Similarity (%)** | **No.**^†^ | **Accession no.** |
| *Actinobacteria* | *Microbacterium flavum* | 99-100 | 12 | ON680767 |
| *Bacteroidetes* | *Chryseobacterium hominis* | 100 | 1 | ON680768 |
| *Bacteroidetes* | *Cloacibacterium caeni* | 100 | 4 | ON680769 |
| *Bacteroidetes* | *Flavobacterium cauense* | 99 | 1 | ON680770 |
| *Firmicutes* | *Bacillus cereus* | 100 | 1 | ON680771 |
| *Firmicutes* | *Lactococcus chungangensis* | 100 | 1 | ON680772 |
| *Alphaproteobacteria* | *Agrobacterium radiobacter* | 100 | 2 | ON680773 |
| *Alphaproteobacteria* | *Allorhizobium borbori* | 100 | 3 | ON680774 |
| *Alphaproteobacteria* | *Novosphingobium stygium* | 97 | 1 | ON680775 |
| *Alphaproteobacteria* | *Paracoccus lutimaris* | 99 | 2 | ON680776 |
| *Alphaproteobacteria* | *Rhizobium arenae* | 98 | 2 | ON680777 |
| *Alphaproteobacteria* | *Rhodobacter thermarum* | 99 | 3 | ON680778 |
| *Alphaproteobacteria* | *Sphingomonas echinoides* | 100 | 6 | ON680779 |
| *Alphaproteobacteria* | *Sphingopyxis terrae* | 100 | 2 | ON680780 |
| *Betaproteobacteria* | *Acidovorax wautersii* | 100 | 3 | ON680781 |
| *Betaproteobacteria* | *Aquitalea aquatilis* | 100 | 1 | ON680782 |
| *Betaproteobacteria* | *Ottowia beijingensis* | 98-100 | 3 | ON680783 |
| *Betaproteobacteria* | *Pelomonas aquatica* | 98 | 4 | ON680784 |
| *Betaproteobacteria* | *Uruburuella suis* | 100 | 1 | ON680785 |
| *Betaproteobacteria* | *Zoogloea caeni* | 97 | 8 | ON680786 |
| *Betaproteobacteria* | *Zoogloea resiniphila* | 99 | 4 | ON680787 |
| *Betaproteobacteria* | *Zoogloea oryzae* | 99 | 3 | ON680788 |
| *Gammaproteobacteria* | *Acinetobacter johnsonii* | 99 | 1 | ON680789 |
| *Gammaproteobacteria* | *Lelliottia jeotgali* | 100 | 1 | ON680790 |
| *Gammaproteobacteria* | *Thermomonas koreensis* | 100 | 1 | ON680791 |
|  |  |  |  |  |
| Species no. | 25 |  |  |  |
| Isolates no. | 71 |  |  |  |
| Species no. / Isolates no. | 0.35 |  |  |  |

^†^The number of isolated colonies

**Table S3 Isolates from soil through the GMD-agg for the co-cultivation test**

| **Label** | **Taxonomic group** | **Closest species** | **Similarity (%)^†^** | **Accession no.** |
| --- | --- | --- | --- | --- |
| G1 | *Alphaproteobacteria* | *Sphingomonas desiccabilis* | 98.98 | PP716552 |
| G2 | *Bacilli* | *Bacillus acidiceler* | 98.81 | PP716553 |
| G3 | *Gammaproteobacteria* | *Dyella marensis* | 99.04 | PP716554 |
| G4 | *Alphaproteobacteria* | *Tardiphaga robiniae* | 97.4 | PP716555 |
| G5 | *Betaproteobacteria* | *Cupriavidus yeoncheonensis* | 99.45 | PP843538 |
| G6 | *Alphaproteobacteria* | *Bradyrhizobium embrapense* | 100 | PP716556 |
| G7 | *Alphaproteobacteria* | *Phenylobacterium hankyongense* | 96.53 | PP716557 |
| G8 | *Alphaproteobacteria* | *Sphingomonas aquatilis* | 94.06 | PP716558 |
| G9 | *Bacilli* | *Bacillus acidiceler* | 97.21 | PP716559 |
| G10 | *Actionobacteria* | *Tetrasphaera japonica* | 97.94 | PP716560 |
| G11 | *Actionobacteria* | *Lysinimicrobium sediminis* | 95.61 | PP716561 |
| G13 | *Actionobacteria* | *Marmoricola pocheonensis* | 97.51 | PP716563 |
| G14 | *Alphaproteobacteria* | *Sphingomonas desiccabilis* | 98.84 | PP716564 |
| G15 | *Betaproteobacteria* | *Burkholderia territorii* | 99.46 | PP716565 |
| G17 | *Flavobateria* | *Flavobacterium ginsenosidimutans* | 96.19 | PP716567 |
| G18 | *Betaproteobacteria* | *Ralstonia pickettii* | 99.06 | PP716568 |
| G19 | *Betaproteobacteria* | *Pelomonas aquatica* | 96.61 | PP716569 |
| G20 | *Betaproteobacteria* | *Pelomonas aquatica* | 97.69 | PP716570 |
| G21 | *Betaproteobacteria* | *Ralstonia pickettii* | 99.73 | PP716571 |
| G22 | *Betaproteobacteria* | *Ralstonia pickettii* | 99.73 | PP716572 |

† Similarity of 16S rRNA between each isolate and its closest species

**Table S4 Isolates from AS through the GMD-agg for the co-cultivation test**

| **Label** | **Taxonomic group** | **Closest species** | **Similarity (%)**^†^ | **Accession no.** |
| --- | --- | --- | --- | --- |
| A2 | *Betaproteobacteria* | *Caldimonas brevitalea* | 97.71 | ON819675 |
| A3 | *Alphaproteobacteria* | *Rhodopseudomonas telluris* | 99.51 | ON819668 |
| A6 | *Gammaproteobacteria* | *Acinetobacter guillouiae* | 98.00 | ON819680 |
| A7 | *Betaproteobacteria* | *Vitreoscilla stercoraria* | 96.92 | ON819676 |
| A11 | *Alphaproteobacteria* | *Brevundimonas albigilva* | 97.17 | ON819661 |
| A12 | *Gammaproteobacteria* | *Acinetobacter vivianii* | 99.03 | ON819686 |
| A13 | *Alphaproteobacteria* | *Sphingomonas aquatilis* | 99.52 | ON819669 |
| A14 | *Actinobacteria* | *Microbacterium hominis* | 98.27 | ON819649 |
| A15 | *Gammaproteobacteria* | *Acinetobacter seifertii* | 99.84 | ON819684 |
| A17 | *Betaproteobacteria* | *Acidovorax wautersii* | 99.84 | ON819670 |
| A24 | *Gammaproteobacteria* | *Acinetobacter chengduensis* | 98.56 | ON819679 |
| A25 | *Betaproteobacteria* | *Herbaspirillum aquaticum* | 100 | ON819672 |
| A28 | *Alphaproteobacteria* | *Rhizobium pakistanense* | 97.91 | ON819667 |
| A29 | *Gammaproteobacteria* | *Acinetobacter tandoii* | 99.67 | ON819685 |
| A30 | *Betaproteobacteria* | *Duganella rivi* | 100 | ON819671 |
| A31 | *Alphaproteobacteria* | *Allorhizobium borborid* | 100 | ON819658 |
| A33 | *Actinobacteria* | *Rhodococcus qingshengii* | 100 | ON819653 |
| A37 | *Gammaproteobacteria* | *Acinetobacter junii* | 99.20 | ON819682 |
| A38 | *Betaproteobacteria* | *Aquitalea pelogenes* | 100 | OP020674 |
| A39 | *Actinobacteria* | *Microbacterium oxydans* | 100 | ON819652 |
| A48 | *Gammaproteobacteria* | *Stenotrophomonas terrae* | 99.03 | ON819688 |
| A56 | *Alphaproteobacteria* | *Novosphingobium lentum* | 97.42 | ON819665 |
| A64 | *Alphaproteobacteria* | *Bosea lupini* | 99.19 | ON819660 |
| A66 | *Gammaproteobacteria* | *Acinetobacter beijerinckii* | 98.26 | ON819683 |
| A69 | *Alphaproteobacteria* | *Brevundimonas terrae* | 99.19 | ON819662 |

† Similarity of 16S rRNA between each isolate and its closest species
